# Unique Features of a Japanese ‘*Candidatus* Liberibacter asiaticus’ Strain Revealed by Whole Genome Sequencing

**DOI:** 10.1371/journal.pone.0106109

**Published:** 2014-09-02

**Authors:** Hiroshi Katoh, Shin-ichi Miyata, Hiromitsu Inoue, Toru Iwanami

**Affiliations:** 1 NARO Institute of Fruit Tree Science, Tsukuba, Ibaraki, Japan; 2 Kuchinotsu Citrus Research Station, NARO Institute of Fruit Tree Science, Minami-shimabara, Nagasaki, Japan; Belgian Nuclear Research Centre SCK•CEN, Belgium

## Abstract

Citrus greening (huanglongbing) is the most destructive disease of citrus worldwide. It is spread by citrus psyllids and is associated with phloem-limited bacteria of three species of α-Proteobacteria, namely, ‘*Candidatus* Liberibacter asiaticus’, ‘*Ca.* L. americanus’, and ‘*Ca.* L. africanus’. Recent findings suggested that some Japanese strains lack the bacteriophage-type DNA polymerase region (DNA pol), in contrast to the Floridian psy62 strain. The whole genome sequence of the pol-negative ‘*Ca.* L. asiaticus’ Japanese isolate Ishi-1 was determined by metagenomic analysis of DNA extracted from ‘*Ca.* L. asiaticus’-infected psyllids and leaf midribs. The 1.19-Mb genome has an average 36.32% GC content. Annotation revealed 13 operons encoding rRNA and 44 tRNA genes, but no typical bacterial pathogenesis-related genes were located within the genome, similar to the Floridian psy62 and Chinese gxpsy. In contrast to other ‘*Ca.* L. asiaticus’ strains, the genome of the Japanese Ishi-1 strain lacks a prophage-related region.

## Introduction

Citrus greening (huanglongbing) is a devastating citrus disease that affects crops around the world. The disease was first noted in China in the early 20^th^ century [1]. Three species of phloem-limited, gram-negative bacteria in the genus ‘*Candidatus* Liberibacter’ are associated with greening. ‘*Ca.* L. africanus’ is mainly present in Africa [2]; ‘*Ca.* L. americanus’ is found in Brazil [3]. A third species, ‘*Ca.* L. asiaticus’ is particularly widespread in Asian countries as well as in Sao Paulo, Brazil and Florida, USA. ‘*Ca.* L. asiaticus’ is transmitted by phloem-feeding insect vectors, the Asian citrus psyllid *Diaphorina citri* [4] and the African citrus psyllid *Trioza erytreae* [5]. A new Liberibacter species, ‘*Ca.* L. solanacearum’, was recently associated with the emerging ‘zebra chip’ disease of potatoes in the U.S. and tomatoes in New Zealand [6].

Little is known about the genetic diversity of ‘*Ca.* L. asiaticus’; the bacteria are difficult to culture, although some successes have been reported [7], [8], [9]. Diversity studies of ‘*Ca.* L. asiaticus’ have been restricted to the 16S/23S rRNA genes, the *omp* gene region, the *rplKAJL-rpoBC*, *nus*G-*rpl*K operon sequence, or bacteriophage-type DNA polymerase region (DNA pol) [10]–[20]. However, the complete genomic sequence of the pathogenic ‘*Ca.* L. asiaticus’ Floridian strain “psy62” (1.23 Mb) [21] has been determined, thus enabling genome-wide analysis. In fact, Chen et al. characterized variation in “*Ca.* L. asiaticus” strains by using one repeat unit (AGACACA) [22]. From the whole-genome sequence, we selected 25 simple sequence repeat loci, including one repeat unit reported by Chen et al. [22] and successfully differentiated ‘*Ca.* L. asiaticus’ strains using these SSR loci [23], [24]. Zhou et al. identified two hypervariable genes in the prophage regions of the psy62 genome [25]. Morgan et al. improved real-time PCR detection of ‘*Ca.* L. asiaticus’ from citrus and psyllid hosts by using the prophage gene [26]. The whole-genome sequencing of ‘*Ca.* L. asiaticus’ Floridian psy62 strain significantly advanced the study of diversity in this species.

Zhang et al. [27] reported two highly related, circular bacteriophage-type genes associated with ‘*Ca.* L. asiaticus’, named SC1 and SC2. Both were found integrated into the ‘*Ca.* L. asiaticus’ Floridian UF506 strain genome as prophages [27]. SC1 was apparently a fully functional, temperate phage with a lytic cycle that was seemingly activated when its host bacterium was present in plants but not when in psyllids [27]. SC2 replicates as an excision plasmid when its ‘*Ca.* L. asiaticus’ host is present in either plants or psyllids [27]. These findings suggest the bacteriophage-type genes are important for infection and virulence expression. However, most of the Japanese ‘*Ca.* L. asiaticus’ strains lack the bacteriophage-type DNA polymerase gene [18], [19]. In Floridian UF506, the bacteriophage-type DNA polymerase gene is flanked by SC1 and SC2. Thus, absence of the bacteriophage-type DNA polymerase gene in Japanese strains suggests they also lack SC1 and SC2. Thus, the Japanese strains have unique genomic features.

In contrast to ‘*Ca.* L. asiaticus’ Floridian strains psy62 and UF506, the whole genome sequence of a Japanese ‘*Ca.* L. asiaticus’ strain lacking the bacteriophage-type DNA polymerase gene has not been reported. Recently, the complete genome sequence of the Chinese ‘*Ca.* L. asiaticus’ strains gxpsy [28] and A4 [29] were reported, although the latter remains in the draft form. Both Chinese ‘*Ca.* L. asiaticus’ strains also contained the bacteriophage-type DNA polymerase gene. The results encouraged us to perform whole-genome sequencing of a Japanese strain lacking this gene. Duan et al. [21] obtained a complete circular ‘*Ca.* L. asiaticus’ Floridian psy62 strain genome by metagenomic analysis of DNA extracted from a single ‘*Ca.* L. asiaticus’-infected psyllid. We used a similar method to obtain the complete genome of the uncultured ‘*Ca.* L. asiaticus’ Japanese strain Ishi-1.

## Materials and Methods

### Bacterial strains

**Figure 1:**
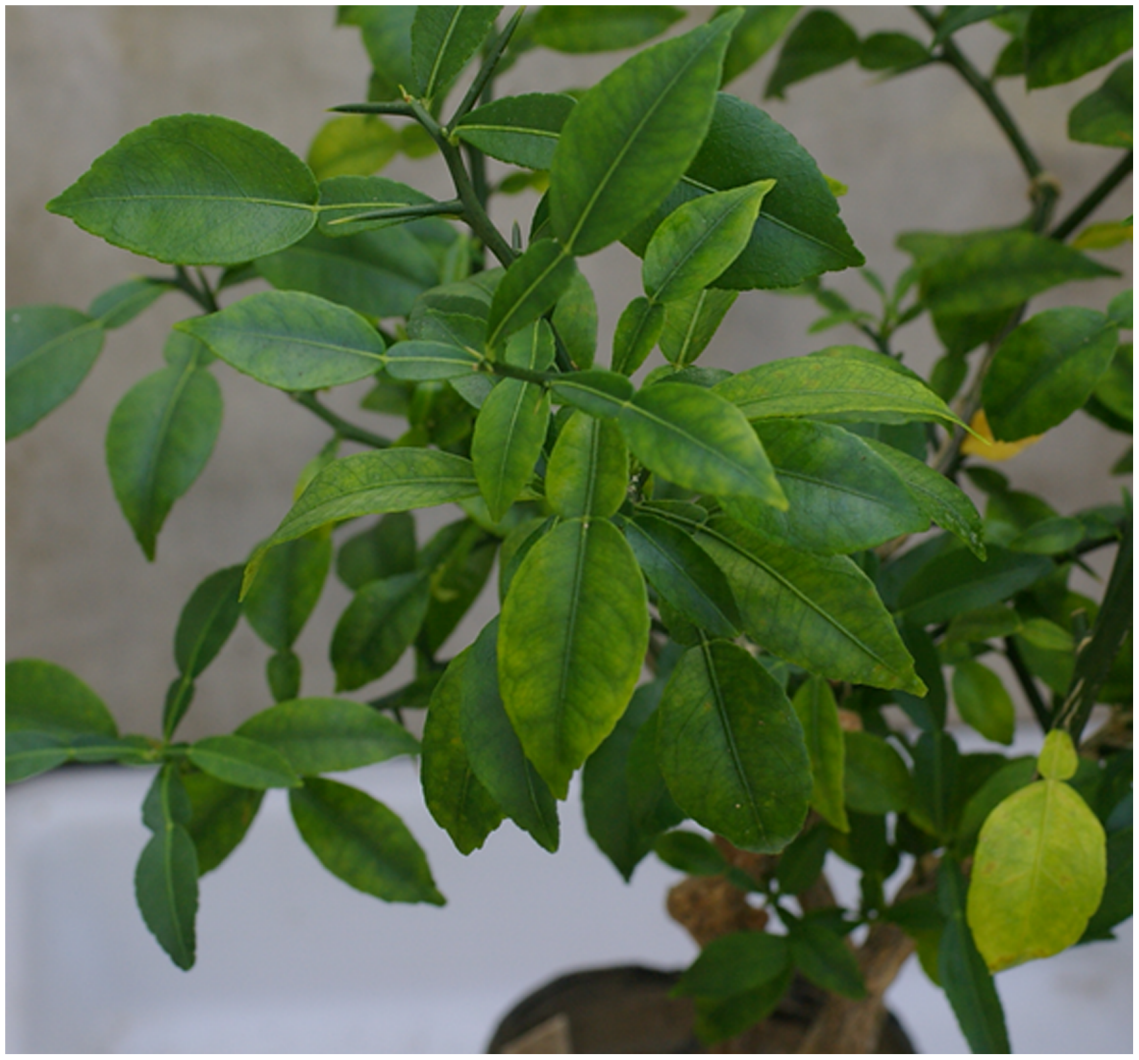
Foliar symptoms on Yuzu (*Citrus junos* Tanaka) induced by ‘*Ca.* L. asiaticus’ Japanese Ishi-1. Severe yellowing on the leaves of a Yuzu plant kept in a closed chamber at the NARO Institute of Fruit Tree Science.

Japanese ‘*Ca.* L. asiaticus’ strain Ishi-1 was used throughout the study. The strain was originally found in local citrus of unidentified cultivars on Ishigaki Island, Okinawa prefecture, Japan. The infected scion was sent to the NARO Institute of Fruit Tree Science (NIFTS) with permission from the plant quarantine office of Japan, and kept in the isolated greenhouse after grafting on rough lemon (*Citrus jambhiri* Lush) rootstocks. The strain Ishi-1 induced severe symptoms on rough lemon, yuzu (*Citrus junos* Tanaka, Figure 1) and other citrus cultivars.

### Psyllid treatment

All experiments using live individuals of *D. citri* were performed in insect-proof growth chambers at 25°C with a 16L∶8D photoperiod at the Kuchinotsu Citrus Research Station, NIFTS (Otsu 954, Kuchinotsu, Minamishimabara, Nagasaki 859–2501, Japan). Healthy fifth instars of psyllids were transferred to an HLB-affected rough lemon tree (*Citrus jambhiri*, approximately 40 cm in height) with a high titer of ‘*Ca.* L. asiaticus’ bacteria. After acquisition feeding for 20 days on the infected plant, nine emerged adults were reared individually for 20 days on healthy *Citrus junos* seedlings for incubating the HLB bacteria, and they were stored at −50°C.

### DNA extraction and quantitative real-time PCR

Total DNA was purified from the entire body of single psyllids using the DNeasy Blood and Tissue Kit (Qiagen, Tokyo, Japan) and a plastic homogenizer pestle (As One, Tokyo, Japan) according to the manufacturer's instructions, and eluted in 150 µL.

Individual psyllid DNA samples were analyzed for ‘*Ca.* L. asiaticus’ populations by quantitative real-time PCR analysis as described by Inoue et al. [30]. Samples containing copies of ‘*Ca.* L. asiaticus’ genomic DNA were selected by real-time PCR (data not shown). Whole-genome amplification was performed with Illustra GenomiPhi V2 (GE Healthcare, Buckinghamshire, England) according to the manufacturer's instructions. DNA concentration was estimated with the Qubit 2.0 instrument and the Qubit dsDNA HS Assay (Life Technologies, Invitrogen, California).

### Genome sequencing and mapping

Sequencing was performed at the Bio Dragon Genomics Center (Takara Bio Co. Ltd. Mie, Japan). DNA libraries with 300∼350 bp inserts were constructed according to manufacturer's instructions (Illumina GaIIx platform) and 75-bp paired-end reads were generated on an Illumina HiSeqTM 2000 platform. Reads were mapped to the ‘*Ca.* L. asiaticus’ Floridian psy62 genome using BWA [31] and Bowtie [32]. Mapping results were visualized with Integrative Genomics Viewer (IGV) version 2.3 [33].

### Polymerase chain reaction for whole genome mapping confirmation

After initial genome mapping results were obtained, ambiguous sequences were determined by PCR amplification and conventional sequencing on an ABI 3130*×l* instrument. Total DNA was extracted from the leaf midrib tissue of citrus trees infected with the ‘*Ca.* L. asiaticus’ Japanese Ishi-1 strain. Total DNA was extracted with the DNeasy plant minikit (Qiagen, Valencia, CA) according to manufacturer's instructions with minor modifications: approximately 0.2 g of the leaf midrib was placed in 400 µL AP1 buffer in a mortar and ground with a pestle until the leaf midrib became a fine green liquid.

Many In/Dels and SNPs were found by mapping the sequence reads of Ishi-1 to the complete sequence of the pathogenic ‘*Ca.* L. asiaticus’ Floridian psy62 (1.23 Mb) strain, and primers were designed from the surrounding sequences (Primer3, http://frodo.wi.mit.edu/primer3/) (Table S1). Other primers were selected from Duan et al. [21]. PCR was performed with the Gene Amp PCR System 9700 (Applied Biosystems, Foster City, CA) in 20-µl reactions containing 1 µl DNA template, 0.1 µM each primer, 200 µM dNTPs, 1× PCR buffer, and 2.5 units of *Ex Taq* DNA polymerase Hot Start Version (TaKaRa, Shiga, Japan) under the following cycling conditions: initial denaturation at 92°C for 2 min; 35 cycles of denaturation at 92°C for 30 s, annealing at 54°C for 30 s, and extension for 1 min/kb of the desired product at 72°C. Long-range PCR of products above 3.0 kbp was performed with Tks Gflex DNA polymerase (TaKaRa). Each 50-µl reaction contained 1 µl DNA template, 0.1 µM each primer, 2× Gflex PCR buffer (Mg^2+^, dNTP plus), and 1.25 U Tks Gflex DNA polymerase. Cycling conditions were as follows: 30 cycles of denaturation at 98°C for 10 s, annealing at 52°C for 15 s, and extension for 30 s/kb of the desired product at 68°C.

DNA sequences were aligned using GENETYX-windows ver. 11 (Software Development, Tokyo, Japan), and homology analysis was performed as recommended by the DNA Data Bank of Japan (http://www.ddbj.nig.ac.jp/Welcome-j.html).

### Gene prediction and functional annotation

Gene prediction and functional annotation were performed with the Microbial Genome Annotation Pipeline (MiGAP) (http://www.migap.org/index.php/en, [34]). Detection of tRNAs and rRNA was performed with tRNAScan-SE 1.23 (http://lowelab.ucsc.edu/tRNAscan-SE/, [35]) and RNAmmer 1.2 (http://www.cbs.dtu.dk/services/RNAmmer/, [36]).

## Results

### Whole genome re-sequencing

**Figure 2:**
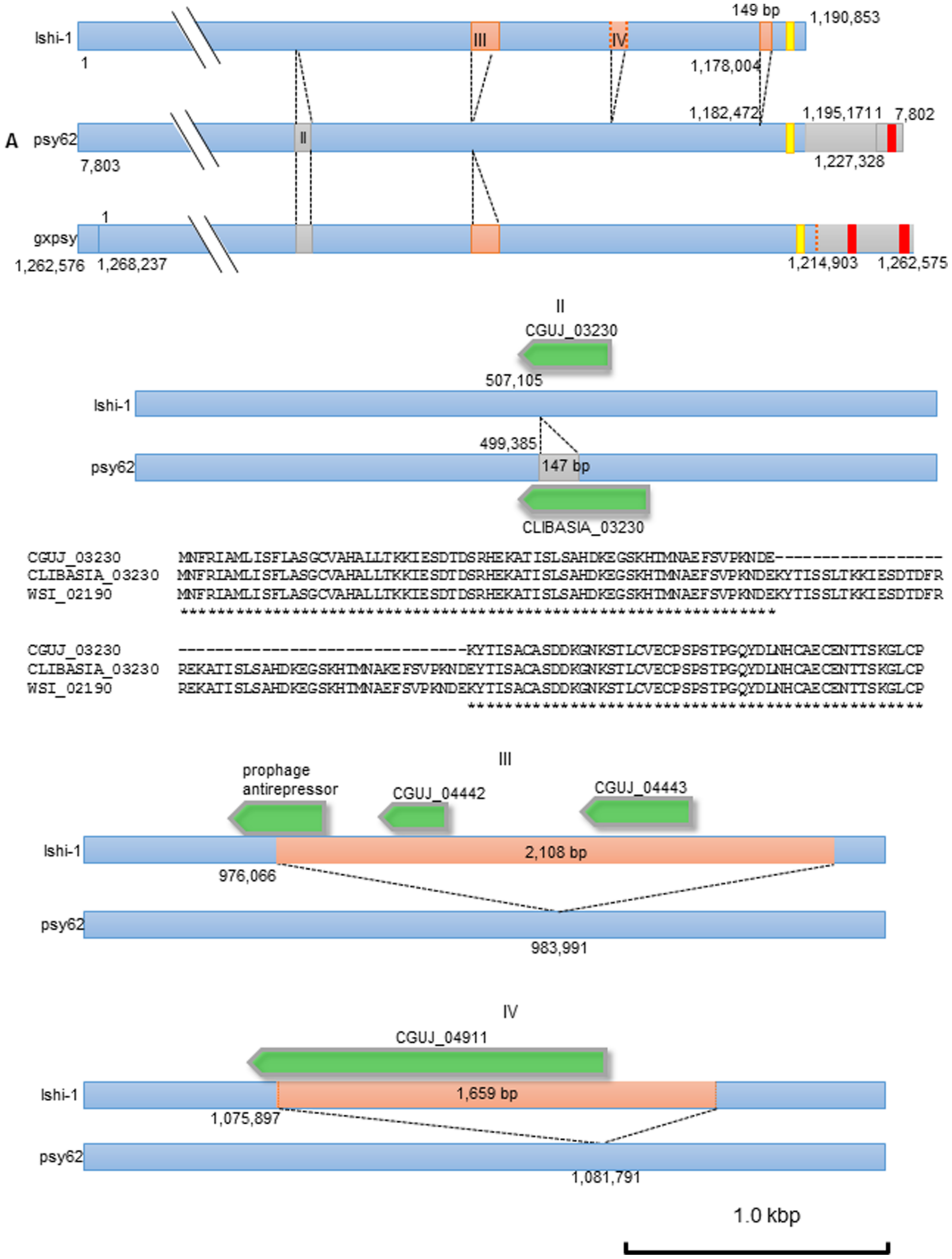
Whole-genome comparison of ‘*Ca.* L. asiaticus’ Floridian psy62 and Japanese Ishi-1. A, Schematic linear alignment between ‘*Ca.* L. asiaticus’ Floridian psy62 and Japanese Ishi-1. Orange/gray boxes (designated I, II, III, and IV) represent four large insertion/deletion domains in ‘*Ca.* L. asiaticus’ Japanese Ishi-1. Other In/Del and SNP variants are ignored. Vertical dotted lines in domain IV in the Ishi-1 box indicate the unclear insertion borders. The number by each box indicates the nucleotide position of each strain. **B, C, and D,** Enlarged maps of domains II, III, and IV in Figure 3 A. Green arrows indicate CDS. B, Deduced amino acid sequences of the hypothetical protein at CLIBASIA_03230 of psy62, WSI_02190 of gxpsy, and CGUJ_03230 of Ishi-1 aligned by CLUSTAL W [48] and identical residues are indicated with asterisks. Databank accession numbers are CP001677 for psy62 [21], AP014595 for Ishi-1, and CP004005 for gxpsy [28].

Sequencing yielded 2,721,927,150 bp of DNA from 36,292,362 pair-end reads of 75 bp. As a result of mapping using BWA, Bowtie against the psy62 strain reference, reads of 14.6% of the 2.7 Gbp were mapped. Coverage to the reference was 96.9%. The sequence reads of Ishi-1 were not mapped near the nucleotide position of 0 to 7,803 and after nucleotide position 1,195,171 of the linear genomic map of psy62, indicating that Ishi-1 lacks a large genomic fragment. PCR amplification of the Ishi-1 template with the LJ754r and LJ764f primers, which are separated by about 35 kbp on the psy62 sequence, yielded a 2.6-kbp product. Sequence analysis showed that the 2.6 kbp fragment filled the 35-kbp gap in the Ishi-1genome. The results clearly demonstrated that Ishi-1 lacks about 33 kbp, corresponding to both ends of the linear genomic map of psy62 (Figure 2). Likewise, by mapping the candidate SNPs/InDels and PCR verification, the draft genome of Ishi-1 was obtained.

**Figure 3:**
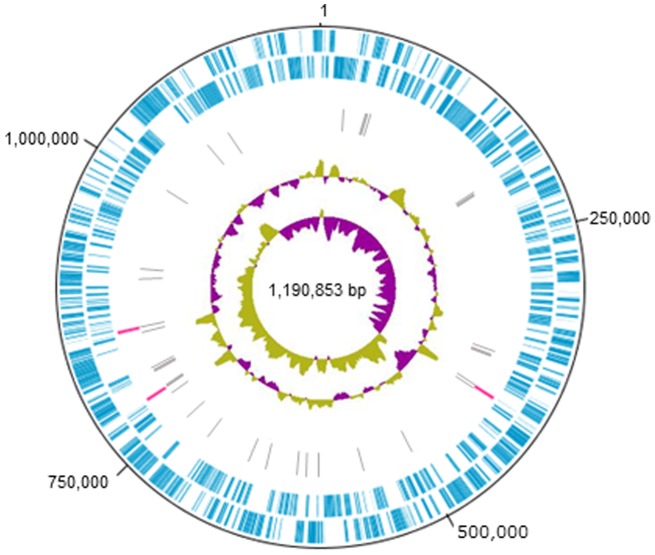
Schematic representation of the genome of ‘*Ca.* L. asiaticus’ Japanese Ishi-1. Circular representation of the 1.19 Mbp genome. The tracks from the outmost circles represent (1) Forward CDS (blue) and (2) Reverse CDS (blue); (3) six copies of the rRNA operon (16S, 23S and 5S) (pink); (4) tRNA (gray): (5)% G+C content (yellow-green, purple), and (6) GC skew [(G−C/(G+C))] (yellow-green, purple).

In order to confirm the whole genome sequence of Ishi-1 strain, six primers published by Duan [21] were selected, and 128 new primers were designed for conventional and long PCR (Table S1). All amplicons generated from these primers were directly sequenced on an ABI 3130*×l*. In total, we re-sequenced over 40,000 bp by Sanger sequencing. These efforts generated a circular chromosome sequence consisting of 1,190,853 bp (Figure 3).

### General features of the ‘*Ca.* L. asiaticus’ Japanese Ishi-1 genome

The calculated GC content of the Ishi-1 genome is 36.32%, similar to other ‘*Ca.* L. asiaticus’ strains (Table 1). After annotation, the newly confirmed CDS regions were compared to those of other ‘*Ca.* L. asiaticus’ strains. Then, the tRNAs and rRNA of Japanese Ishi-1 were compared to those of Floridian psy62. These analyses revealed 1,075 coding sequences and 975 ribosome binding sites. We also found 44 tRNA genes that were shared with the Floridian psy62 and Chinese gxpsy strains, as well as 13 rRNA operons and 313 hypothetical proteins (Table 1). Our comparison of ‘*Ca.* L. asiaticus’ Japanese Ishi-1 and Floridian psy62 revealed 291 base substitutions (Table 2). We also confirmed 122 in/del loci (Table 2). The five SSR loci were also polymorphic between the two strains (Table 2).

**Table 1:** Comparison of the whole genome among three strains of ‘*Ca.* Liberibacter asiaticus’ and ‘*Ca.* L. solanacearum.’

Features	‘*Ca.* L. asiaticus’			‘*Ca.* L. solanacearum’
	Ishi-1^a^	psy62^b^	gxpsy^c^	Clso-ZC1^d^
Size(bp)	1,190,853	1,227,328	1,268,237	1,258,278
GC%^e^	36.3	36.5	36.5	35.2
rRNA operons	13	9	6	9
tRNA	44	44	44	45
RBS	975	1022	1078	1093
CDS	1075	1134	1165	1192
hypothetical protein	313	358	368	409

a Accession number AP014595.

b Accession number CP001677 [21].

c Accession number CP004005 [28].

d Accession number NC_014774 [37].

e GC contents were calculated using GENETYX ver. 11.

**Table 2:** Base substitution, insertion, deletion mutation and repeat number at respective SSR motif between ‘*Ca.* L. asiaticus’ Japanese Ishi-1 and Floridian psy62 strain.

	Ishi-1	psy62	SSR motif	Ishi-1	psy62
Base substitution	291	–	TACAGAA	14	8
Insertion (one base insertion)	66 (58)	–	AGACACA	8	5
Deletion (One base deletion)	56 (38)	–	TTTG	9	14
			TTTTAA	5	3
			AGA	6	5

### Bacteriophage-type polymerase and other genes

As described above, the biggest difference in Japanese Ishi-1 is the absence of the 33-kbp fragment (Figure 3 A). In psy62, this fragment encodes 40 CDS, including the bacteriophage-type polymerase gene between the SC1 and SC2 genes in the prophage region (Table 3). Most of the 40 CDS are shared between psy62, UF506, and Gxpsy (Table 3). None of these 40 CDS, including the bacteriophage-type polymerase gene, were found elsewhere in the genome of Ishi-1. Thus, Ishi-1 lacks the bacteriophage-type DNA polymerase gene found in Floridian strains psy62 and UF506, and the Chinese gxpsy strain ([21], [27], [28], shown by a vertical red line in Figure 3A). Another bacteriophage-type DNA polymerase is encoded in the middle of the linear schematic representation of the psy62 genome (shown by a vertical yellow line in Figure 3A). This bacteriophage-type DNA polymerase is also encoded in the corresponding region of the Ishi-1 genome (Figure 3A). Thus, it became clear that Ishi-1 carries a single bacteriophage-type DNA polymerase gene, whereas psy62 has two. In contrast, Chinese gxpsy and Floridian UF506 carry three bacteriophage-type DNA polymerase genes (WSI_05345, 05570, 05770, UF506_015, SC1_gp210, SC2_gp210).

**Table 3:** List of CDS encoded in the large 33 kbp fragment that is retained by Floridian psy62, UF506 and Chinese gxpsy strains, but not by Japanese Ishi-1 strain of ‘*Ca.* L. asiaticus.’

psy62			UF506			gxpsy		
product	locus tag	Locus location in genome^a^	product	locus tag	Locus location in genome^b^	product	locus tag	Locus location in genome^c^
hypothetical protein^d^	CLIBASIA_00005	36..407	hypothetical protein	SC1_gp185 SC2_gp185	76449..76820 36401..36772	hypothetical protein	WSI_05545	1214938..1215309
hypothetical protein	CLIBASIA_00010	497..820	hypothetical protein	SC1_gp190 SC2_gp190	76910..77233 36862..37185	hypothetical protein	WSI_05550 WSI_05750	1215399..1215722 1255266..1255589
hypothetical protein	CLIBASIA_00015	948..2114	hypothetical protein	SC1_gp195 SC2_gp195	77361..78527 37313..38479	hypothetical protein	WSI_05555 WSI_05755	1215850..1217016 1255717..1256883
prophage antirepressor	CLIBASIA_00020	2285..3073	putative Bro-N family phage antirepressor	SC1_gp200 SC2_gp155	78698..79486 32107..32292	prophage antirepressor	WSI_05560 WSI_05760	1217187..1217978 1257054..1257845
hypothetical protein	CLIBASIA_00025	3091..3741	hypothetical protein	SC1_gp205 SC2_gp205	79504..80154 39456..40106	hypothetical protein	WSI_05565 WSI_05765	1217996..1218646 1257863..1258513
putative DNA polymerase from bacteriophage origin	CLIBASIA_00030	3745..5772	DNA polymerase A	SC1_gp210 SC2_gp210	80158..82185 40110..42137	putative DNA polymerase from bacteriophage origin	WSI_05570 WSI_05770	1218650..1220677 1258517..1260544
VRR-NUC domain-containing protein	CLIBASIA_00035	5769..6080	endonuclease	SC1_gp215 SC2_gp215	82182..82493 42134..42445	VRR-NUC domain-containing protein	WSI_05575 WSI_05775	1220674..1220985 1260541..1260852
hypothetical protein	CLIBASIA_00040	6065..6727	SNF2 Dead box helicase	SC2_gp220	42430..43815	SNF2 related protein	WSI_05580 WSI_05780	1220970..1222355 1260837..1262222
DNA ligase, NAD-dependent	CLIBASIA_00050	7442..7801	DNA ligase	SC2_gp225 SC1_gp225	4811..5170 43808..44167	DNA ligase, NAD-dependent	WSI_05360 WSI_05585 WSI_05785	1183651..1184010 1222348..1222707 1262215..1262574
guanylate kinase	CLIBASIA_05525	1195911..1196264	hypothetical protein	SC1_gp235	44890..45261	guanylate kinase	WSI_05595	1223448..1223780
hypothetical protein	CLIBASIA_05530	complement(1196268..1196741	hypothetical protein	SC1_gp005	complement(45265..45738)	hypothetical protein	WSI_05600	complement(1223784..1224257)
hypothetical protein	CLIBASIA_05531	complement(1196738..1197010)	hypothetical protein	SC1_gp010	complement(45735..46007)	hypothetical protein	WSI_05410	complement(1188796..1189068)
hypothetical protein	CLIBASIA_05538	complement(1197003..1199762)	hypothetical protein	SC1_gp025	complement(46325..48448)	hypothetical protein	WSI_05610	complement(1224519..1226903)
hypothetical protein	CLIBASIA_05545	complement(1199769..1202363)	hypothetical protein	SC1_gp030	complement(48455..51049)	hypothetical protein	WSI_05615	complement(1226910..1229504)
hypothetical protein	CLIBASIA_05550	complement(1202360..1203796)	hypothetical protein	SC1_gp035	complement(51046..52482)	hypothetical protein	WSI_05620	complement(1229501..1230937)
hypothetical protein	CLIBASIA_05555	complement(1203814..1205937)	hypothetical protein	SC1_gp045	complement(52500..54623)	hypothetical protein	WSI_05625	complement(1230955..1233078)
hypothetical protein	CLIBASIA_05560	complement(1205934..1206449)	hypothetical protein	SC1_gp050	complement(54620..55123)	hypothetical protein	WSI_05630	complement(1233075..1233596)
hypothetical protein	CLIBASIA_05565 CLIBASIA_05570	complement(1205934..1206449) complement(1210209..1210451)	hypothetical protein	SC1_gp060	complement(55116..59138)	hypothetical protein	WSI_05635	complement(1233577..1237620)
hypothetical protein	CLIBASIA_05575	complement(1210476..1212212)	hypothetical protein	SC1_gp080	complement(59163..60899)	hypothetical protein	WSI_05640	complement(1237645..1239381)
hypothetical protein	CLIBASIA_05580	complement(1212205..1212732)	hypothetical protein	SC1_gp085	complement(60892..61419)	hypothetical protein	WSI_05645	complement(1239374..1239901)
hypothetical protein	CLIBASIA_05585	complement(1212732..1213763)	putative major capsid protein	SC1_gp090 SC2_gp090	complement(61419..62450) complement(22025..22945)	hypothetical protein	WSI_05455	complement(1200642..1201673)
hypothetical protein	CLIBASIA_05590	complement(1213776..1214480)	hypothetical protein	SC1_gp095	complement(62463..63167)	hypothetical protein	WSI_05655	complement(1240893..1241594)
hypothetical protein	CLIBASIA_05595	complement(1214491..1214820)	hypothetical protein	SC1_gp100	complement(63178..63507)	hypothetical protein	WSI_05660	complement(1241605..1241934)
head-to-tail joining protein, putative	CLIBASIA_05600	complement(1214813..1216483)	putative phage-related head-to-tail joining protein	SC1_gp105	complement(63500..65170)	head-to-tail joining protein, putative	WSI_05665	complement(1241927..1243597)
hypothetical protein	CLIBASIA_05605	complement(1216480..1216812)	hypothetical protein	SC1_gp110	complement(65167..65499)	hypothetical protein	WSI_05670	complement(1243594..1243926)
putative phage terminase, large subunit	CLIBASIA_05610	complement(1216885..1218420)	putative phage terminase, large subunit	SC1_gp115	complement(65572..67107)	putative phage terminase, large subunit	WSI_05680	complement(1244335..1245870)
hypothetical protein	CLIBASIA_05615	complement(1218677..1218793)				hypothetical protein	WSI_05480 WSI_05685	complement(1206133..1206249) complement(1246127..1246243)
hypothetical protein	CLIBASIA_05620	complement(1218955..1219443)	hypothetical protein	SC1_gp120 SC2_gp120	complement(67642..68157) complement(27687..28202)	hypothetical protein	WSI_05690	complement(1246405..1246893)
hypothetical protein	CLIBASIA_05625	complement(1220547..1221164)	hypothetical protein	SC1_gp125	complement(69236..69853)	hypothetical protein	WSI_05695	complement(1247997..1248614)
hypothetical protein	CLIBASIA_05630	1221334..1221936	hypothetical protein	SC1_gp130	70104..70625	hypothetical protein	WSI_05700	1248784..1249386
hypothetical protein	CLIBASIA_05635	1221998..1222390	hypothetical protein	SC1_gp135 SC2_gp135	70687..71079 30619..31011	hypothetical protein	WSI_05500 WSI_05705	1209271..1209663 1249448..1249840
hypothetical protein	CLIBASIA_05640	1222526..1222732	hypothetical protein	SC1_gp140 SC2_gp140	71215..71421 31147..31353	hypothetical protein	WSI_05505 WSI_05710	1209800..1210006 1249977..1250183
hypothetical protein	CLIBASIA_05645	1222725..1222937	hypothetical protein	SC1_gp145 SC2_gp145	71414..71626 31346..31558	hypothetical protein	WSI_05510 WSI_05715	1209999..1210211 1250176..1250388
intrrupted gp228, phage associated protein	CLIBASIA_05650	1222969..1223301	hypothetical protein e	SC1_gp150 SC2_gp150	71658..72062 31590..31994	intrrupted gp229, phage associated protein	WSI_05515 WSI_05720	1210243..1210575 1250420..1250752
hypothetical protein	CLIBASIA_05655	complement(1223555..1223866)	hypothetical protein	SC1_gp160 SC2_gp160	complement(72591..72968) complement(32523..32900)	hypothetical protein	WSI_05725	complement(1251006..1251317)
P4 family phage/plasmid primase	CLIBASIA_05660	complement(1223914..1226283)	phage associated primase	SC1_gp165	complement(73016..75388)	P4 family phage/plasmid primase	WSI_05730	complement(1251365..1253734)
hypothetical protein	CLIBASIA_05665	complement(1226284..1226673)	hypothetical protein	SC1_gp170 SC2_gp170	complement(75389..75778) complement(35321..35710)	hypothetical protein	WSI_05535 WSI_05735	complement(1213869..1214258) complement(1253735..1254124)
hypothetical protein	CLIBASIA_05670	complement(1226691..1226897)	hypothetical protein	SC1_gp175 SC2_gp175	complement(75796..76002) complement(35728..35934)	hypothetical protein	WSI_05540 WSI_05740	complement(1214276..1214482) complement(1254142..1254348)
hypothetical protein	CLIBASIA_05675	complement(1226894..1227157)	hypothetical protein	SC2_gp180	complement(35931..36194)			

a Data are based on the genome sequence of ‘*Ca.* L. asiaticus’ Floridian psy62 strain. The accession number is CP001677 [21].

b Data are based on the genome sequence of ‘*Ca.* L. asiaticus’ Floridian UF506 strain. The accession number is HQ377374 [27].

c Data are based on the genome sequence of ‘*Ca.* L. asiaticus’ Chinese gxpsy strain. The accession number is CP004005 [28].

d Products on the same line indicates that the deduced amino acid sequences has a huge similarity to one of ‘*Ca.* L. asiaticus’ Floridian psy62 strain on the far left.

e Underline revealed that the deduced amino acid sequences showed about 80% similarity to one of ‘*Ca.* L. asiaticus’ Floridian psy62 strain on the far left.

Absence of the 33 kbp-fragment means other genes are also missing from the Ishi-1 genome. For example, Ishi-1 carries two NAD-dependent DNA Ligase genes (CGUJ_05395, 05515), whereas psy62 carries three (CLIBASIA_00050, 05395, 05515)—as does Chinese gxpsy (WSI_05360, 05585, 05785). In addition, the putative phage terminase, large subunit, exists in a single copy (CGUJ_05470) in the genome of Ishi-1, but as two copies in psy62 (CLIBASIA_05470, 05610), and Chinese gxpsy carries three (WSI_05315, 05475, 05680). Furthermore, the genome of Ishi-1 does not contain a full-length P4 family phage/plasmid primase gene; psy62 carries one (CLIBASIA_05660) and the Chinese gxpsy genome carries two (WSI_05530, 05730).

### Characteristics of ‘*Ca.* L. asiaticus’ Japanese Ishi-1 strain marked by large In/Del variations

Several large In/Dels are shown in the simplified schematic presentation of the genome (Figure 3 A). The 147-bp deletion at nucleotide positions 507106 through 507252 of the Floridian psy62 strain was detected in the genome of ‘*Ca.* L. asiaticus’ Japanese Ishi-1 (Figure 3 B). This deletion reduced the hypothetical protein sequence at CGUJ_03230 by 49 amino acids in comparison to CLIBASIA_03230 of psy62 and WSI_02190 of Chinese gxpsy (Figure 3 B). In contrast, the 2,108 bp insertion between nucleotide positions 983990 and 983991 (Figure 3 C), an **untranslated region** in psy62, was detected in Ishi-1. This insertion carries a prophage anti-repressor at CGUJ_04441, and two hypothetical proteins at CGUJ_04442 and CGUJ_04443 were newly confirmed. The deduced amino acid sequence of the prophage anti-repressor at CGUJ_04441 was identical to that of Chinese gxpsy (WSI_04270), but different from those of Floridian psy62 and UF506. The hypothetical protein at CGUJ_04442 was also identical to that of Chinese gxpsy (WSI_04275). The hypothetical protein at CGUJ_04443 locus shared 99% amino acid sequence identity with the putative WSI_04280 in Chinese gxpsy. These two hypothetical proteins shared no identity with any of the hypothetical proteins from Floridian psy62 and UF506.

Another insertion around nucleotide position 1081791 (psy62) was detected in Ishi-1 (Figure 3 D), encoding a hypothetical protein at CGUJ_04911 within the 1,660-bp span. The deduced amino acid sequence of the hypothetical protein shares 48% identity with the hypothetical protein CKC_03455 from ‘*Ca.* L. solanacearum’ CLso-ZC1, a pathogen of zebra chip. Ishi-1 also carries a 149 bp-long insertion that correspond to the nucleotide positions 1182471 and 1182472 of psy62 (Figure 3A). No reading frames were found in the insertion.

### Other In/Del and non-synonymous SNPs affecting annotation of ‘*Ca.* L. asiaticus’ Japanese Ishi-1

**Figure 4:**
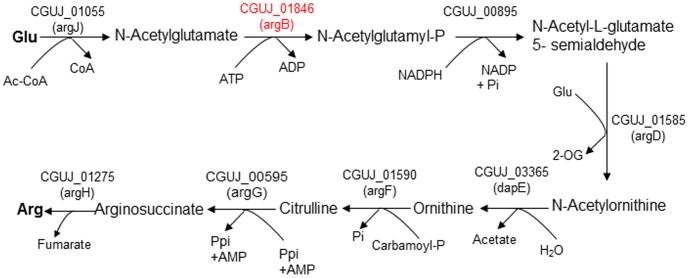
Analysis of the ‘*Ca.* L. asiaticus’ Japanese Ishi-1 arginine biosynthesis pathway. The typical prokaryotic arginine biosynthesis pathway. The NAGK family of enzymes catalyze the second step of arginine biosynthesis and are known as argB in many bacteria [44], [45], [46], [47]. The argB that was not encoded by ‘*Ca.* L. asiaticus’ Floridian psy62 but encoded by Japanese Ishi-1 and Chinese gxpsy is indicated in red letters.

Lin et al. noted the absence of a full-length *N*-acetylglutamate kinase (NAGK) in the genome of ‘*Ca.* L. asiaticus’ Floridian psy62, although it is present in ‘*Ca.* L. solanacearum’ CLso-ZC1 [37]. However, Japanese Ishi-1 (CGUJ_01846) and Chinese gxpsy (WSI_01760, [28]) encode identical full-length NAGK. Within the three ‘*Ca.* L. asiaticus’ strains, psy62 lacks an adenine between 406695 and 406696, thus truncating the sequence. The presence of an NAGK coding sequence indicates that Ishi-1 has a complete pathway for the production of arginine from glutamine, unlike psy62 (Figure 4).

Because of a single base insertion, Ishi-1 has two copies of the malic enzyme gene at CGUJ_00080 and CGUJ_00081, while ‘*Ca.* L. asiaticus’ Floridian psy62 (CLIBASIA_00080) and Chinese gxpsy (WSI_00005) each carry a single copy.

The genome of Ishi-1 also encodes a non-heme ferritin-like protein (CGUJ_03035), just like psy62 [38], [39]. This ferritin-like protein is also found in ‘*Ca.* L. solanacearum’ [37], but is absent from the genomes of all other *Rhizobiaceae*. The ferritin superfamily of proteins includes several diverse members that are typically involved in iron storage and detoxification [40], [41], [42]. Lin et al. hypothesized that this ferritin-like protein may play a critical role in the survival and/or virulence of ‘*Ca.* L. solanacearum’ and ‘*Ca.* L. asiaticus’ [37]. The genome of ‘*Ca.* L. asiaticus’ Chinese gxpsy also encodes a ferritin-like protein (WSI_2370). These sequences were identical but for a single amino acid substitution in Japanese Ishi-1 (Figure S1). In contrast, Floridian UF506 does not encode ferritin, indicating that this protein is dispensable.

Lin et al. reported that the ‘*Ca.* L. solanacearum’ genome encodes three known proteins involved in DNA replication and repair, all of which are absent from ‘*Ca.* L. asiaticus’ Floridian psy62: LexA, DnaE, and RadC [37]. The genome Japanese Ishi-1 does not encode LexA, but does encode DnaE at CGUJ_03631 and RadC at CGUJ_03976. Chinese gxpsy also encodes DnaE and RadC, at WSI_03515 and WSI_03815, respectively (Table 4). However, a nucleotide sequence identical to that encoding RadC on the other ‘*Ca.* L. asiaticus’ strains is also carried in psy62 (Table 4), while LexA and DnaE are absent. This difference might be an annotation error.

**Table 4:** Presence of deduced amino acid sequence related DNA replication.

	LexA		DnaE		RadC	
	locus tag	nucleotide position	locus tag	nucleotide position	locus tag	nucleotide position
Ishi-1	-	-	CGUJ_03631	789771..793445	CGUJ_03976	ccomplement(866926..867195)
psy62	-	-	-	-	-	874850..875119
gxpsy	-	-	WSI_03515	782257..785931	WSI_03815	complement(859383..859676)
Clso-ZC1	CKC_02355	507699..508370	CKC_05200	complement(1116198..1119878)	CKC_04675	986531..987244

-: not identified.

The hypothetical protein at CGUJ_03991 was newly confirmed because of a one-base substitution in the untranslated region of the psy62 genome. The hypothetical proteins shared no similarity to other proteins of ‘*Ca.* L. asiaticus’. These proteins are listed in Table S2.

## Discussion

As described previously, ‘*Ca.* L. asiaticus’ Japanese Ishi-1 lacks a bacteriophage-type DNA polymerase gene [19]. Our study showed that Ishi-1 lacks a large fragment of about 33 kbp that contains the bacteriophage-type DNA polymerase gene. It is noteworthy that this strain is found only in Japan; despite having the smallest genome of all ‘*Ca.* L. asiaticus’ strains, Floridian UF506 carries the large 33-kbp fragment [27]. We suggest the large 33-kbp fragment is associated with neither pathogenicity nor transmissibility, because Ishi-1 induced severe symptoms on citrus and propagated to a high titer in the vector insect. This is in sharp contrast to the discussions of Zhang et al. [27] regarding UF506, where the SC1 and SC2 genes flanking the bacteriophage-type DNA polymerase gene are suspected to be important for infection and virulence expression. It is likely that Ishi-1 carries different virulence factors. Most Japanese strains also lack the bacteriophage-type DNA polymerase gene [19]. Thus, the large 33-kbp fragment encoding the bacteriophage-type DNA polymerase gene may be absent from other Japanese strains, although confirmation by sequencing is needed. Another bacteriophage-type DNA polymerase is encoded in the middle of the Floridian psy62 and Japanese Ishi-1 genomes (Figure 3A), while Chinese gxpsy and Floridian UF506 carry two additional polymerases. These differences also suggest Ishi-1 (and perhaps other Japanese strains) are distinct from other strains from the US and China.

Zhou et al. identified two related and hypervariable genes (*hyv*_I_ and *hyv*_II_) in the large 33-kbp fragment of the psy62 genome [25]. Although all DNA samples were obtained from symptomatic tissue and tested positive by 16S rRNA gene-based real-time PCR, neither the *hyv*_I_ nor the *hyv*_II_ gene was amplified from eight Indian citrus DNA samples and six Philippine psyllid DNA samples using the same primer sets [25]. These 14 strains likely lack the large 33-kbp fragment as Japanese Ishi-1 does. Thus, ‘*Ca.* L. asiaticus’ lacking the large fragment are not limited to Japan but are widespread in South Asia, pending confirmation by genome sequencing. We conclude it is best not to use primer sets specific to the large 33 kbp fragment for PCR detection of ‘*Ca.* L. asiaticus’, because some strains might escape detection.

Two malic enzyme genes were identified in the genome sequence of Ishi-1 in contrast to ‘*Ca.* L. asiaticus’ Floridian psy62 and Chinese gxpsy. The malic enzyme of the microaerophilic protist *Entamoeba histolytica* decarboxylates malate to pyruvate [43]. In ‘*Ca.* L. asiaticus’, a phloem-limited bacterium [2], the enzyme might play a similar role to that of *E. histolytica*. However, both malic enzyme genes of Ishi-1 are shorter than those of other ‘*Ca.* L. asiaticus’ strains. The Ishi-1 enzymes might not maintain their original function, suggesting a possible limitation of the Ishi-1 fermentation pathway.

In conclusion, whole genome sequencing of Japanese ‘*Ca.* L. asiaticus’ strain Ishi-1 revealed unique genomic features and suggested novel expression of virulence and establishment in host plant as well as distinct molecular evolution. We hope this study will advance our understanding of ‘*Ca.* L. asiaticus’ and facilitate efforts to control this devastating disease in the citrus industry.

## Supporting Information

Figure S1 Comparison of deduced amino acid sequences of ferroxidase from ‘*Ca.* L. asiaticus’. Ferroxidase sequences were aligned by CLUSTAL W [48], and identical residues are indicated with asterisks. Databank accession numbers are CP001677 for psy62 [21], AP014595 for Ishi-1, and CP004005 for gxpsy [28], respectively.
(TIF)

Table S1 Conventional and long-distance PCR primers used for ‘Ca. L. asiaticus’ Japanese Ishi-1 strain sequence confirmation and gap closeure in this study.
(XLSX)

Table S2 List of CDS in the genome of ‘Candidatus Liberibacter asiaticus’ Japanese Ishi-1 strain that revealed no deduced amino acid sequence similarity to those of other ‘Ca. L. asiaticus’ strains.
(XLSX)
